# Mixed Phenolic Acids Mediated Proliferation of Pathogens *Talaromyces helicus* and *Kosakonia sacchari* in Continuously Monocultured *Radix pseudostellariae* Rhizosphere Soil

**DOI:** 10.3389/fmicb.2016.00335

**Published:** 2016-03-17

**Authors:** Hongmiao Wu, Linkun Wu, Juanying Wang, Quan Zhu, Sheng Lin, Jiahui Xu, Cailiang Zheng, Jun Chen, Xianjin Qin, Changxun Fang, Zhixing Zhang, Saadia Azeem, Wenxiong Lin

**Affiliations:** ^1^Fujian Provincial Key Laboratory of Agroecological Processing and Safety Monitoring, Fujian Agriculture and Forestry UniversityFuzhou, China; ^2^Key Laboratory of Biopesticide and Chemical Biology, Ministry of Education, Fujian Agriculture and Forestry UniversityFuzhou, China; ^3^Key Laboratory of Crop Ecology and Molecular Physiology, College of Life Sciences, Fujian Agriculture and Forestry UniversityFuzhou, China

**Keywords:** *Radix pseudostellariae*, monoculture cropping problem, phenolic acids, *Talaromyces helicus*, *Kosakonia sacchari*, *Bacillus pumilus*

## Abstract

*Radix pseudostellariae* L. is a common and popular Chinese medication. However, continuous monoculture has increased its susceptibility to severe diseases. We identified two pathogenic microorganisms, *Talaromyces helicus* M. (KU355274) and *Kosakonia sacchari* W. (KU324465), and their antagonistic bacterium, *Bacillus pumilus* Z. in rhizosphere soil of continuously monocultured *R. pseudostellariae*. Nine types of phenolic acids were identified both in the rhizosphere soil and in culture medium under sterile conditions. A syringic acid and phenolic acid mixture significantly promoted the growth of *T. helicus* and *K. sacchari*. *T. helicus* could utilize eight types of phenolic acids, whereas *K. sacchari* could only use four phenolic acids. *K. sacchari* produced protocatechuic acid when consuming vanillin. Protocatechuic acid negatively affected the growth of *B. pumilus*. The 3A-DON toxin produced by *T. helicus* promoted the growth of *K. sacchari* and inhibited growth of *B. pumilus* at low concentrations. These data help explain why phenolic exudates mediate a microflora shift and structure disorder in the rhizosphere soil of continuously monocultured *R. pseudostellariae* and lead to increased replanting disease incidence.

## Introduction

*Radix pseudostellariae* L. (*Caryophyllaceae*) is a common and popular Chinese medicine. It contains polysaccharides, ginseng saponins, flavonoids, cyclic peptides, amino acids, and trace elements (Hang and Wang, 2010). High-quality *R. pseudostellariae* is mainly produced in the ZheRong region of Fujian province in southern China, where soil and climate conditions are favorable for its growth. However, continuously monocultured *R. pseudostellariae* is prone to severe diseases, which result in reduced biomass of the plant tuberous roots (Lin et al., 2012a,b; Zhao et al., 2015). This phenomenon is known as replanting disease or soil sickness. It has been reported that more than 70% of medicinal plants, especially those with tuberous roots, such as *R. pseudostellariae*, have been attacked by replanting diseases (Zhang and Lin, 2009). Therefore, it has become the priority to study and overcome the consecutive monoculture problems, especially exhibited in medicinal plant production.

Replanting disease (Wu et al., 2009; Zhang and Wang, 2010), or “Sick Soil Syndrome,” is a problem related to replanting in soils where the same species was previously grown. The sick soil syndrome refers to a combination of plant growth dysplasia, pests, and disease problems, which result in reduced yield and quality. It is a consequence of continuous monocropping for many years. Autotoxicity is an intraspecific allelopathy phenomenon, common in monocropping systems, where the plants inhibit their own growth through release of autotoxic chemicals into the soil (Singh et al., 1999; Wu et al., 2015). Terpenoids, phenolics, steroids, alkaloids, and cyanogenic glycosidic exudates are secreted from monocultured crop plant roots (Kato-Noguchi et al., 2002; Kato-Noguchi and Ino, 2003; Kulmatiski et al., 2008). Phenolic acids are important in allelopathy and replanting disease incidence (Bais et al., 2004; Kulmatiski et al., 2008; Wu et al., 2015; Zhao et al., 2015). Phenolic compounds are autotoxins of the plants in monocropping system, such as *Rehmannia glutinosa*, cucumber, and tobacco (Fang et al., 2010, 2013; Lin et al., 2010; Li et al., 2012; Zhou and Wu, 2012b; Wu et al., 2015). Some studies indicate that phenolic acids change the soil microbial community with possible influences on plant performance (Zhou et al., 2012, 2014). The allelochemicals released by plants might also promote growth of soil-borne pathogens and inhibit beneficial microbes (Kong et al., 2008; Pollock et al., 2011). The details of these phenomena are poorly known, especially in medicinal plants under monoculture regimes.

Most research in crop monocultures has focused on the effects of phenolic acids on the soil microbial community. There is less information on the dynamic changes of phenolics in soil and their effects on specific microbes. Therefore, we identified the types of phenolic acids in soil and their effects on the potential pathogens (*Talaromyces helices* M. and *Kosakonia sacchari* W.) and a beneficial microbe (*Bacillus pumilus* Z.) under laboratory conditions. It was also determined whether the pathogenic microorganisms were able to cause replanting disease in moncultured *R. pseudostellariae*, and the changes were also examined in the soil microbial community structure and its relationship to replanting disease of *R. pseudostellariae* under long term continuous monoculture conditions.

## Materials and methods

### The isolation and identification of pathogenic microorganisms

The pathogenic microorganisms were isolated using a tissue isolation method (Fang et al., 2011). After initial cleaning of the tuber roots of *R. pseudostellariae* sampled from the monoculture plots, we washed them for 30 min under running water, then used sterile water to wash them once on a sterile operation platform. A clean knife was used to cut the pathogen-infected tuber roots into slices. These slices were soaked in 75% ethanol for 30–40 s, washed twice with sterile water, dipped in 0.1% aqueous mercuric chloride solution for 7–10 min and washed again using sterile water (five times, for 2–3 min each time). The sterilized tissue slices of the infected roots were then cultured on MS medium at 32°C, after which the pure strains were separated from the mixed bacterial or fungal community in the culture.

### PCR for bacterial 16S rRNA genes and fungal its regions

PCR amplification of partial bacterial 16S rRNA gene sequences was performed using the primer set 1405f and 456r (Zeng and Zheng, 2011). PCR assays were conducted in a 25-μL volume mix containing 12.5 μL of Taq PCR Master Mix (2 ×) (Transgen, Beijing, China), 1 μL of each primer, and 20 ng of purified DNA extracts. PCR conditions were: 94°C for 5 min; 94°C for 1 min; 61°C for 1 min; 72°C for 90 s, 35 cycles in total, with a final elongation at 72°C for 10 min. The fungal community sizes were estimated using the primer sets ITS4 and ITS86 (Ferrer et al., 2001). The PCR conditions for the fungal community were: 94°C for 5 min; 94°C for 45 s; 51°C for 45 s; and 7°C for 1 min. PCR products were separated by electrophoresis on a 1.2% agarose gel and the bands were purified using the Universal DNA Purification Kit (TIANGEN, Beijing, China). The DNA sequences were analyzed using BLAST tools and the NCBI database to determine the species.

### Validation of microbial pathogenicity

Based on the tissue culture of *R. pseudostellariae*, we added 200 μL of activator fungi, *Kosakonia* and *B. pumilus* (isolated from the rhizosphere of *R. pseudostellariae*) to the tissue culture. The control received equivalent amounts of PDA broth and LB medium instead. Then, the growth of the tissue culture seedlings co-cultured with the specific pathogenic bacteria was observed.

### Validation of *B. pumilus* antagonists against *T. helicus*

The amended agar disk diffusion method (Bauer et al., 1966) was used to qualitatively screen the antagonists. Briefly, two strains of *B. pumilus* were streaked onto a potato dextrose agar (PDA) plate (square plate), and the *Talaromyces helicus* removed from an actively growing colony margin of *T. helicus*, was placed in the center of the plate. Plates were incubated for 8 d at 30°C.

### Quantitative real-time PCR (qPCR) of *T. helicus, K. sacchari*, and *B. pumilus* isolated from the *R. pseudostellariae* rhizosphere soil

According to the sequences of *T. helicus, K. sacchari*, and *B. pumilus*, taxon-specific primers including TH1F/TH1R, saesu-F/saesu-R, and BP-F/BP-R were set (Table S1). The identity and specificity of the primers (the amplifying gene) were confirmed (see Figure S5). Then we used the primers to amplify specific genes through the total DNA extracted from the *R. pseudostellariae* rhizosphere soil. And we also found these primers could amplify the special fragments corresponding to the *T. helicus, K. sacchari*, and *B. pumilus*.

The DNA sequences of *T. helicus, K. sacchari*, and *B. pumilus* were amplified using TH1F/TH1R, saesu-F/saesu-R, and BP-F/BP-R from the plasmid and were purified using a TIAN pure Mini Plasmid Kit [TIANGENBIOTECH (BEIJING) CO., LTD]. The plasmids contained the target DNA which can be represented the different microorganisms. We used these plasmids as standard curves to calculate the contents of *T. helices, K. sacchari*, and *B. pumilus*, respectively. The concentration of the target DNA was determined using a spectrophotometer (NanoDrop2000c, Thermo Fisher, USA), and was diluted to 1, 0.5, 0.1, 0.05, 0.01, 0.005, and 0.001 ng/μL. qPCR was monitored using a Mastercycler ep realplex (Eppendorf, Germany). Standard curve plotting and melting curve analysis was performed following the qPCR amplification instructions. A standard curve was created by plotting the target DNA concentration against the threshold cycle (Ct)-value exported from the Mastercycler ep realplex. The primer sets TH1F/TH1R, saesu-F/saesu-R, and BP-F/BP-R were evaluated using the established standard curve, and melting curves were determined using qPCR amplification of four replicates with a serial dilution of the target DNA template. The qPCR reactions were performed in 15-μL reaction mixtures (7.5 mL 2 × SYBR Green PCR Master Mix, 0.6 μL TH1F/TH1R, 0.6 μL saesu-F/saesu-R, 0.6 μL BP-F/BP-R, and 40 ng DNA made up to a final volume of 15 μL with ddH_2_O). The PCR parameters for *T. helicus* were as follows: 94°C for 5 min, followed by 35 cycles of 94°C for 1 min, 56°C for 45 s, and 72°C for 45 s. The *K. sacchari* PCR parameters were 94°C for 5 min, followed by 35 cycles of 94°C for 1 min, 55°C for 30 s, and 72°C for 30 s, and the *B. pumilus* parameters were 94°C for 5 min, followed by 35 cycles of 94°C for 1 min, 60°C for 45 s, and 72°C for 45 s. After the qPCR run, melting curve analysis was performed to verify the specificity of the amplified product under the following conditions: 95°C for 15 s, 60°C for 15 s, followed by an increase to 95°C over 10 min, and then maintained at 95°C for 15 s.

### Phenolics extraction and determination

#### Soil phenolics extraction

Soil samples were collected from the pots containing *R. pseudostellaria* at different growth stages for each treatment, including consecutive monoculture, newly planted, and control (no plant). Fresh soil was collected on April 22, May 5, June 6, and June 26 in 2014, and the sampled soil was sieved (2-mm mesh) to remove stones and plant residues. Soil phenolic acids of each sample were extracted using previously described methods (Dalton et al., 1987; Zhou and Wu, 2012a). Briefly, 5 g of each soil sample was added to 25 mL of 1 M NaOH and agitated for 24 h on a reciprocal shaker at 30°C, then spun in a vortex generator for 30 min at maximum speed. The suspension was centrifuged at 10,000 rpm for 10 min, and the liquid supernatant was collected. The filtrate was adjusted to pH 2.5 using 9 M HCl, and extracted five times with ethyl acetate. The resultant extracts were pooled and evaporated to dryness at 35°C. The residue was dissolved in 5 mL methanol using ultrasound for 5 min and maintained in the dark at 4°C.

#### Phenolics extraction from the tissue culture medium of *R. pseudostellariae*

The tissue cultures of *R. pseudostellariae* were incubated for 150, 215, 279, 305, and 355 d. Each treatment contained 60 mL of medium. Then, the used medium from each treatment was collected for extraction of the phenolic acids. Each sample was added to 40 mL of 1 M NaOH and agitated for 90 min in an ultrasonic generator. The other conditions were the same as those mentioned above.

#### Phenolics determination

The methanol solution of the extracts was filtered through 0.22-μm filter membrane for HPLC analysis. The phenolic acids in each sample were determined using a Waters HPLC system (C18 column: Inertsil ODS-SP, 4.6 × 250 mm, 5 μm). The mobile phase A was methanol and mobile phase B was 2% glacial acetic acid. The flow rate was kept constant at 0.7 mL/min. Detection was performed at 280 nm. The injection volume was 20 μL and the column temperature was maintained at 30°C. Identification and quantification of phenolic compounds were confirmed by comparing retention times and areas with pure standards.

#### The influence of phenolic acids on *R. pseudostellariae*

Based on the measurement results of the phenolic acids content and the ratio of the mixed phenolic acids detected in *R. pseudostellariae* rhizosphere soil, phenolic acids standards were used to form a “mixed phenolic acid solution (P1)” that simulated the average ratio of various phenolic acids detected in *R. pseudostellariae* rhizosphere soil (FY, SF, TF; i.e., gallic acid: coumaric acid: p-hydroxybenzoic acid: vanillic acid: syringic acid: vanillin: ferulic acid: benzoic acid = 3:11:26: 119:14:14:25:28).

In addition, a series of concentration gradients of the mixed phenolic acids were set at 0, 160, 320, 960, and 1280 μmol/L for the tissue culture of *R. pseudostellariae*. Each treatment had three replicates. Then, the tissue culture was incubated for 50 d. Plant dry mass was measured after oven drying at 70°C to a constant mass.

### The influence of phenolic acids on the physiological characteristics of *T. helices*

#### The impact of single and the mixed phenolic acids on the diameter growth of the *r. pseudostellariae* biotype *T. helicus*

Soil extract-medium (SEM) preparation: 1 kg of soil and 1 L of double distilled water were shaken for 30 min on the shaker, then sterilized at 121°C for 15 min. The leachate was filtered through talc in a double suction filter. The filtrate was collected and adjusted to neutral pH and the mother liquor retained for further use. According to method requirements, double distilled water was used to dilute the soil solution with appropriately diluted replicates, and then an appropriate amount of agar (15 g/L) was added to the solution, which was sterilized before use.

Based on the measurements of phenolic acid content in the *R. pseudostellariae* rhizosphere soil, a series of phenolic acid concentration gradients were set. The concentration gradients were 0, 40, 80, 160, 320, and 960 μmol/L. When the 10-fold dilution of the SEM medium was cooled (to about 40–50°C) after sterilization, appropriate amounts of various stock phenolic solutions were added (dissolved using methanol), after filtration through a 0.22-μm membrane, then immediately poured into the plates. The control received only double distilled water and an equal volume of methanol in order to exclude the effects of methanol on the growth of *T. helices*. Each treatment was replicated three times. After preparation of the phenolic acid-SEM plates, the activated *T. helices* spores were cultured in the center of the plate, then placed in a 30°C constant temperature incubator for 9 d, after which the mycelium diameter was measured.

Based on the phenolic acid content and the ratio of the mixed phenolic acids (P1), a series of phenolic concentrations were established, i.e., 0, 40, 80, 160, 320, and 960 μmol/L. The other conditions were the same as those mentioned above.

#### Effect of the mixed phenolic acids on the toxin production of the *R. pseudostellariae* biotype *T. helicus*

The dilution ratio of the soil filtrate and its preparation were the same as mentioned above. The concentrations of the mixed phenolic acids were 0, 80, 160, and 960 μmol/L. An equal amount of *T. helices* spores filtrate was added to the SEM liquid medium in the bottle. Then, the bottle was maintained at 30°C on a 180-rpm thermostatic shaker for 8 d. After culture, HPLC techniques were used to determine the content of two common toxins (3A-DON: 3-Acetyldeoxynivalenol and 15A-DON: 15-O-Acetyl-4-deoxynivalenol). To draw a standard curve, the concentrations of the two types of toxin standards used were 0.5, 1, 2, 5, and 10 ppm. The chromatographic conditions were as follows: chromatographic column: C18 column (Inertsil ODS-SP, 4.6 × 250 mm, 5 μm); mobile phase A: acetonitrile; mobile phase B: 0.005% phosphoric acid solution; elution gradient: mobile phase B 95% (0 min) → 30% (9 min) → 0% (18 min) → 0% (23 min) → 95% (23.01 min) → 95% (45 min); oven temperature: 35°C; detection wavelength: 224 nm; velocity: 0.7 mL/min.

The extraction method for *T. helices* toxins was as follows. The liquid fermented for 10 d was first filtered using double filter paper and then filtered using a 0.22-μm ultrafiltration membrane. The filtrate (15 mL) was added to a bottle containing the same volume of ethyl acetate, and placed on the shaker for 8 h at 28°C, 160 rpm for oscillation extraction. This was followed by centrifugation at 10,000 rpm for 3 min at 4°C. The upper layer organic phase was transferred into a new 50-mL centrifuge tube. After concentration by vacuum and drying, 500 μL of double evaporated water was added to dissolve the solid residue, then subjected to ultrasound and analyzed immediately using HPLC. The sample toxins were quantitatively analyzed by comparison to the standard curve.

### Detecting the utilization of various phenolic acids by the *R. pseudostellariae* biotype *T. helices*

The soil leachate mother liquid (48 mL) was diluted three times and placed into a 250-mL triangle flask. After sterilization and precooling, 0.3 mL of the phenolic acid mixture (coumaric acid, protocatechuic acid, gallic acid, p-hydroxybenzoic acid, vanillic acid, syringic acid, vanillin, ferulic acid, benzoic acid, 4800 μmol/L) was added to the flask. These phenolic acids had been detected in the rhizosphere soil of *R. pseudostellariae*. Then, 250 μL of *T. helices* spore filtrate was added to the flask. All flasks containing the spores were placed in the constant temperature oscillation shaker at 30°C, 180 rpm. A 1 mL sample of bacterial liquid was collected at 0, 6, 18, 26, 38, 47, 53, 68, 72, and 75 h, filtered through a 0.22-μm ultrafiltration membrane, and then loaded into an HPLC bottle. These samples were analyzed by HPLC using the same chromatographic conditions as previously described.

### Affects of phenolic acids on the physiological characteristics of *K. sacchari*

#### The affects of single and mixed phenolic acids on the growth of *K. sacchari*

The LB liquid culture medium was diluted six times, placed into glass tubes, and subjected to 121°C and high pressure sterilization for 20 min. When the culture medium had cooled, an appropriate amount of each phenolic acid stock solution (such as gallic acid, coumaric acid, p-hydroxybenzoic acid, vanillic acid, syringic acid, vanillin, ferulic acid, benzoic acid), which had passed through a 0.22-μm ultrafiltration membrane and *K. sacchari* liquid (30 μL) that had already been activated were added to each tube. Then, all the tubes, which were maintained at 37°C, were placed on a thermostatic shaker at 200 rpm for 8–10 h. Finally, 200 μL of bacterial liquid was transferred to a 96-well enzyme-labeled plate, and a standard enzyme instrument (Thermo Scientific Multiskan MK3, Shanghai, China) was used to determinate the absorbance values at OD 600 nm.

The LB liquid culture medium was diluted six times and added to an appropriate amount of phenolic acid stock solution P1 previously filtered through a 0.22-μm filtration membrane. The final concentration of each phenolic acid was: 0, 30, 60, 120, 240, 480, and 960 μmol/L. The other conditions were the same as those mentioned above.

#### Detection conditions for phenolic acids used by *K. sacchari*

Soil leachate mother liquor (48 mL) was diluted three times and placed into a 250-mL triangle flask. After sterilization and cooling, eight types of phenolic acids (coumaric acid, gallic acid, p-hydroxybenzoic acid, vanillic acid, syringic acid, vanillin, ferulic acid, and benzoic acid, 4800 μmol/L; 0.3 mL each) were added to the liquor. These phenolic acids were detected in the rhizosphere soil of *R. pseudostellariae*. Then, 50 μL of *K. sacchari* fluid which had been activated, was added to the flask. All the flasks were placed on the oscillation table at a constant 37°C, 180 rpm. From then on, At 0, 3, 6, 9, 12, 15, 26, 29, 32, 35, 38, 41, 48 h, 1 mL samples of bacteria liquid were taken, filtered through 0.22 μm ultrafiltration membrane, and loaded into HPLC bottles. These samples were immediately stored at 4°C or analyzed using HPLC with the same chromatographic conditions as noted earlier.

#### Detection conditions for each single of phenolic acids used by *K. sacchari*

Every triangle flask contained a single phenolic acid, and the culture time was set at 0, 15, 28, 37, and 47 h. The other conditions were the same as those mentioned above.

### Toxin production and autotoxicity bioassay of *K. sacchari* and *B. pumilus*

The LB liquid culture medium was diluted four times and added to an appropriate amount of single toxin (e.g., 3A-DON, 15A-DON), which had been filtered through a 0.22-μm filtration membrane. The final concentrations of each toxin were: 0, 0.005, 0.01, 0.02, 0.04, 0.08, 0.16, and 0.32 mg/L. Bacterial liquid (60 μL), previously activated, was added to each tube. All tubes were maintained at 37°C on a thermostatic shaker at 200 rpm for 7 h. Finally, bacterial liquid (200 μL) was placed in a 96-well enzyme-labeled plate, and a standard enzyme instrument was used to determinate the absorbance values at OD 600 nm.

### The effect of the reaction intermediate, protocatechuic acid, on the beneficial microorganism *B. pumilus*

The concentrations of protocatechuic acid were: 0, 30, 60, 120, 240, 480, and 960 μmol/L. *B. pumilus* liquid (60 μL), previously activated, was added to each tube. The other conditions were the same as those mentioned above.

### Statistical analysis

Differences among the treatments were calculated and statistically analyzed using the analysis of variance (ANOVA) and the LSD multiple range test (*p* < 0.05). The Statistical Package for the GraphPad Prism version 5.1 and the Data Processing System (DPS) version 7.05 were used for statistical analysis.

## Results

### Identification of microorganisms and validation of their pathogenicity

The DNA sequences were analyzed by means of BLAST tools and the NCBI database. The detected microorganisms were *K. sacchari* (KU324465) and *T. helices* (KU355274) which were highly pathogenic to the tissue culture plantlets of *R. pseudostellariae* (Figures S1, S2). However, *B. pumilus* Z. was not pathogenic to the tissue culture plantlets (Figure S3) and suppressed the mycelial growth of *T. helicus* when it was co-cultured with the pathogen (Figure S4).

### Dynamics of *T. helicus, K. sacchari*, and *B. pumilus* in the rhizosphere soil of *R. pseudostellariae*

**Figure 1:**
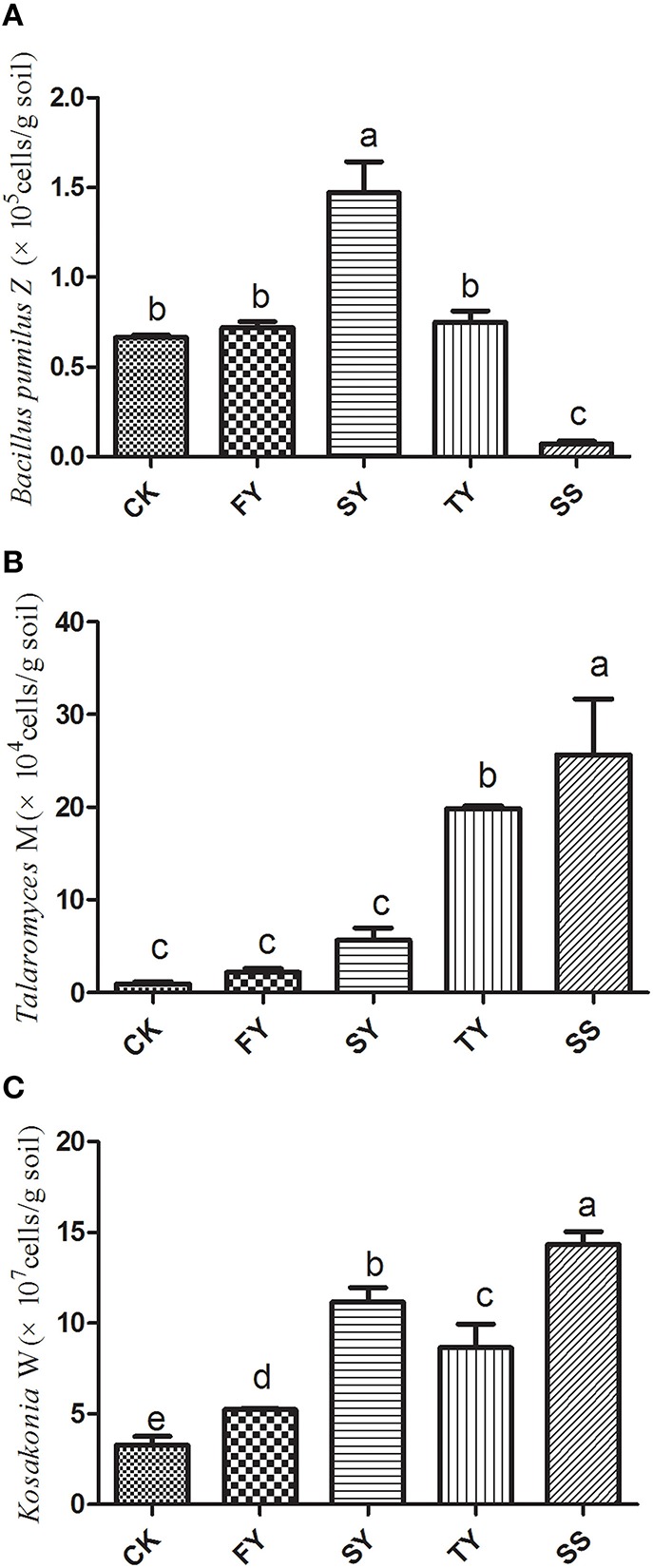
**The content of *Bacillus pumilus* Z (A), *Talaromyces* M (B), and *Kosakonia* W (C) in *R. pseudostellariae* rhizosphere soil after a different number of years of monoculture**. FY, SY, TY represent the first, second, and third cropping of *R. pseudostellariae*, respectively, grown in pots with the soil from the same plot; SS represents the soil around the pathogenic site of *R. pseudostellariae*. Columns with different letters are statistically different (LSD-test, *p* < 0.05).

The qPCR analysis showed a significant increase in the amount of pathogenic *T. helicus* and *K. sacchari* in the rhizosphere of *R. pseudostellariae* as the number of monoculture years increased, especially around the site of infected *R. pseudostellariae* (SS). This is consistent with the observation that soil-borne diseases become more severe with an increase in the number of monoculture years. However, *B. pumilus* showed the opposite trend, which tended to increase in 2-year monoculture soil and then significantly decrease in the 3-year monoculture soil (Figure 1).

### Component identification and analysis of phenolic acid content in *R. pseudostellariae* rhizosphere soil and tissue culture medium

**Figure 2:**
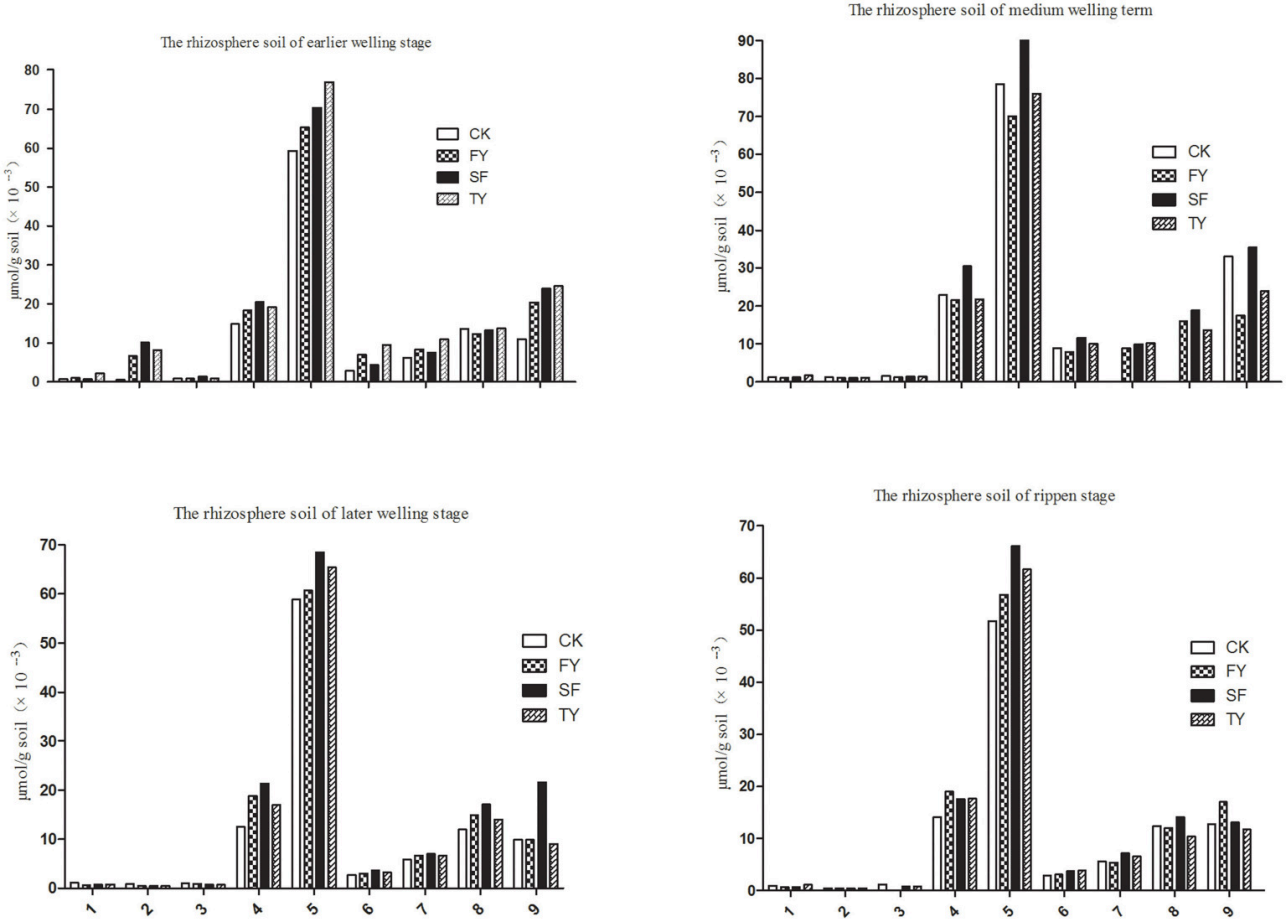
**Changes in the levels of phenolic compounds in the rhizosphere soil of *R. pseudostellariae* in a continuous cropping system sampled at different growth stages**. 1, Represents gallic acid; 2, represents coumaric acid; 3, represents protocatechuic acid; 4, represents p-hydroxybenzoic acid; 5, represents vanillic acid; 6, represents syringic acid; 7, represents vanillin; 8, represents ferulic acid; 9, represents benzoic acid; FY, SY, TY: represent the first, second, and third cropping of *R. pseudostellariae*, respectively, grown in pots with the soil from the same plot.

**Figure 3:**
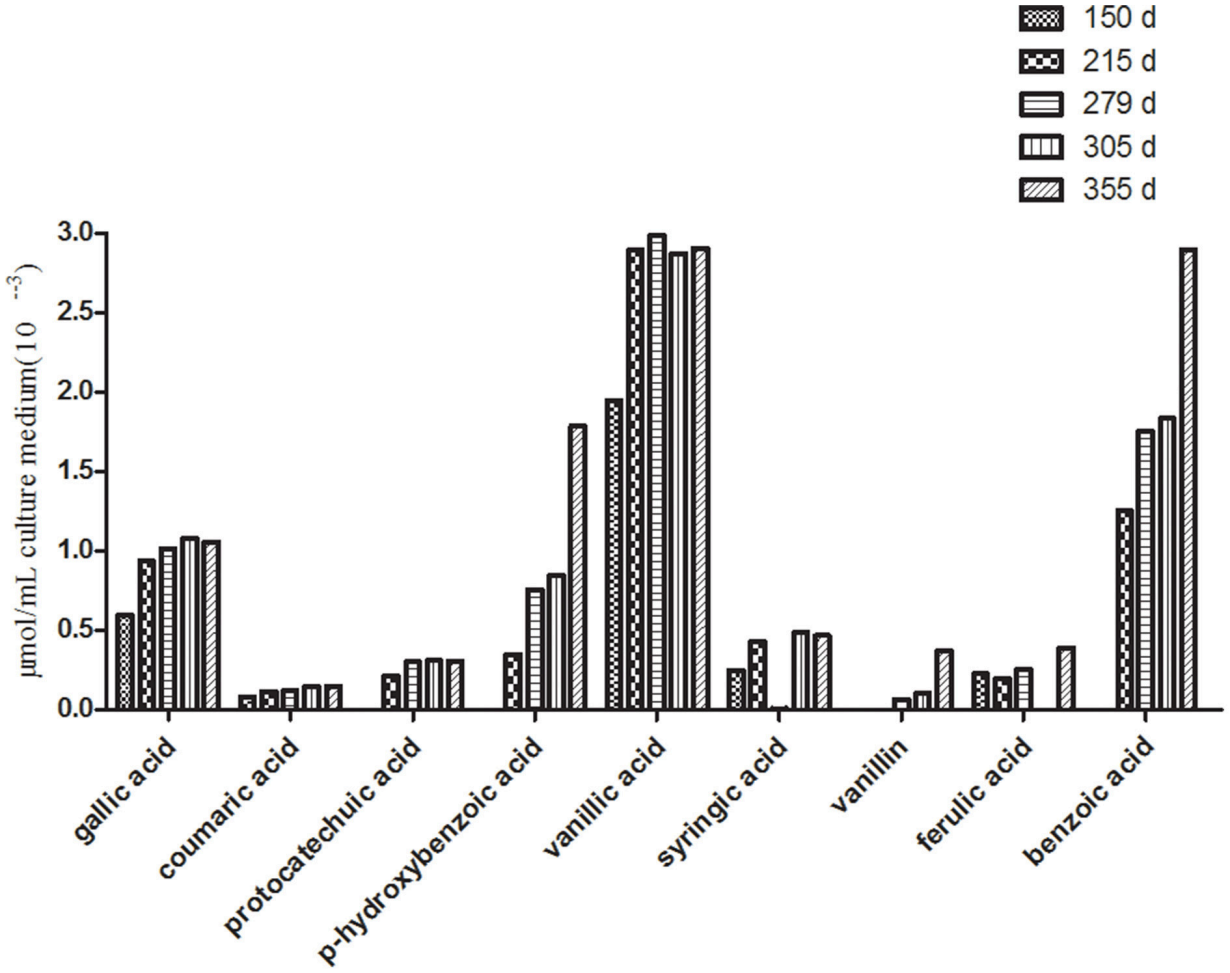
**Changes in the contents of the phenolic compounds in the tissue culture medium of *R. pseudostellariae* on different growth days**.

HPLC was used to determine phenolic acids in the rhizosphere soil of *R. pseudostellariae* at different growth stages in different years of monoculture. We identified nine types of phenolic acids in soil. These were gallic acid, coumaric acid, protocatechuic acid, p-hydroxy benzoic acid, vanillic acid, syringic acid, vanillin, ferulic acid, and benzoic acid as shown in Figure 2, Figure S6, and Table 1. We also found these phenolic acids in the tissue culture medium of *R. pseudostellariae* (Figure 3).

**Table 1:** **Regression equation and detectable limitations**.

**Components**	**Regression equation**	**Correlation coefficient (*R*^2^)**	**LOD (μg/L)**	**LOQ (μg/L)**
Gallic acid	*Y* = 6.829175 * 10^−5^*x* − 5.005725 * 10^−2^	0.9995360	0.1	1
Coumaric acid	*Y* = 2.405028 * 10^−5^*x* + 7.812873 * 10^−3^	0.9999587	0.8	5
Protocatechuic acid	*Y* = 2.234748 * 10^−5^*x* + 1.787522 * 10^−2^	0.9999678	0.8	10
P-hydroxybenzoic acid	*Y* = 2.457867 * 10^−5^*x* − 4.562858 * 10^−2^	0.9973118	0.2	2
Vanillic acid	*Y* = 2.346689 * 10^−5^*x* + 1.073886 * 10^−2^	0.9999876	0.2	1
Syringic acid	*Y* = 1.310058 * 10^−5^*x* + 7.292201 * 10^−3^	0.9999619	0.05	1
Vanillin	*Y* = 9.395554 * 10^−6^*x* + 1.664172 * 10^−2^	0.9999803	0.1	1
Ferulic acid	*Y* = 1.254746 * 10^−5^*x* − 1.618195 * 10^−2^	0.9999014	0.2	2
Benzoic acid	*Y* = 9.88553*10^−5^*x* + 0.235101	0.9996993	1	10
Salicylic acid	*Y* = 6.11749 * 10^−5^*x* + 0.2245029	0.9995034	1	10
3A-DON	*A* = 2.518697 * 10^−5^*x* − 0.1851545	0.9997099	5	50
15A-DON	*A* = 5.06982 * 10^−5^*x* + 0.01643754	0.9995085	10	100

*Y, means the concentration of phenolic; x, means the peak area; A, means the concentration of toxins*.

HPLC analysis showed that the phenolic acid levels in the monocultured rhizosphere soil varied with different plant growth stages. For example, most of the phenolic acids fluctuated. They increased initially and then declined (Table 2 and Figure S7). However, phenolic acid levels tended to increase in the culture medium as the growth of the tissue culture plantlets increased. Gallic acid, P-hydroxybenzoic acid vanillic acid, and benzoic acid significantly accumulated in the culture medium (Figure 3). These phenolic acids did not show autotoxicity toward the culture plantlets of *R. pseudostellariae* (Figure S8). This implies that microorganisms might be involved in the variability of phenolic acid levels in monocultured rhizosphere soil.

**Table 2:** **The dynamic changes of phenolic acids in the rhizosphere soil of *R. pseudostellariae* at different growth stages in a continuous cropping system**.

**Growth stage**	**The changing trend of phenolic acid**			
	**↑**	**∧**	**∨**	**↓**
Earlier welling stage	Gallic acid, vanillic acid, ferulic acid, benzoic acid	Protocatechuic acid, p-hydroxy benzoic acid	Coumaric acid, syringic acid, vanillin	
Medium welling stage	Gallic acid, vanillin	protocatechuic acid, p-hydroxy benzoic acid, vanillic acid, syringic acid, ferulic acid, benzoic acid		Coumari-c acid
Later welling stage	Gallic acid, coumaric acid	P-hydroxybenzoic acid, vanillic acid, syringic acid, vanillin, ferulic acid, benzoic acid		Protocat-echuic acid
Ripening stage	Gallic acid, syringic acid, protocatechuic acid	Coumaric acid, vanillic acid, vanillin, ferulic acid	P-hydroxybenzoic acid	Benzoic acid

*↑, Means an increasing trend; V, means an initial declining trend followed by an increase, whereas, ∧, means an increasing trend followed by a decline; ↓, means a declining trend*.

### The influence of phenolic acids on the physiological characteristics of the *R. pseudostellariae* biotype *T. helices*

**Figure 4:**
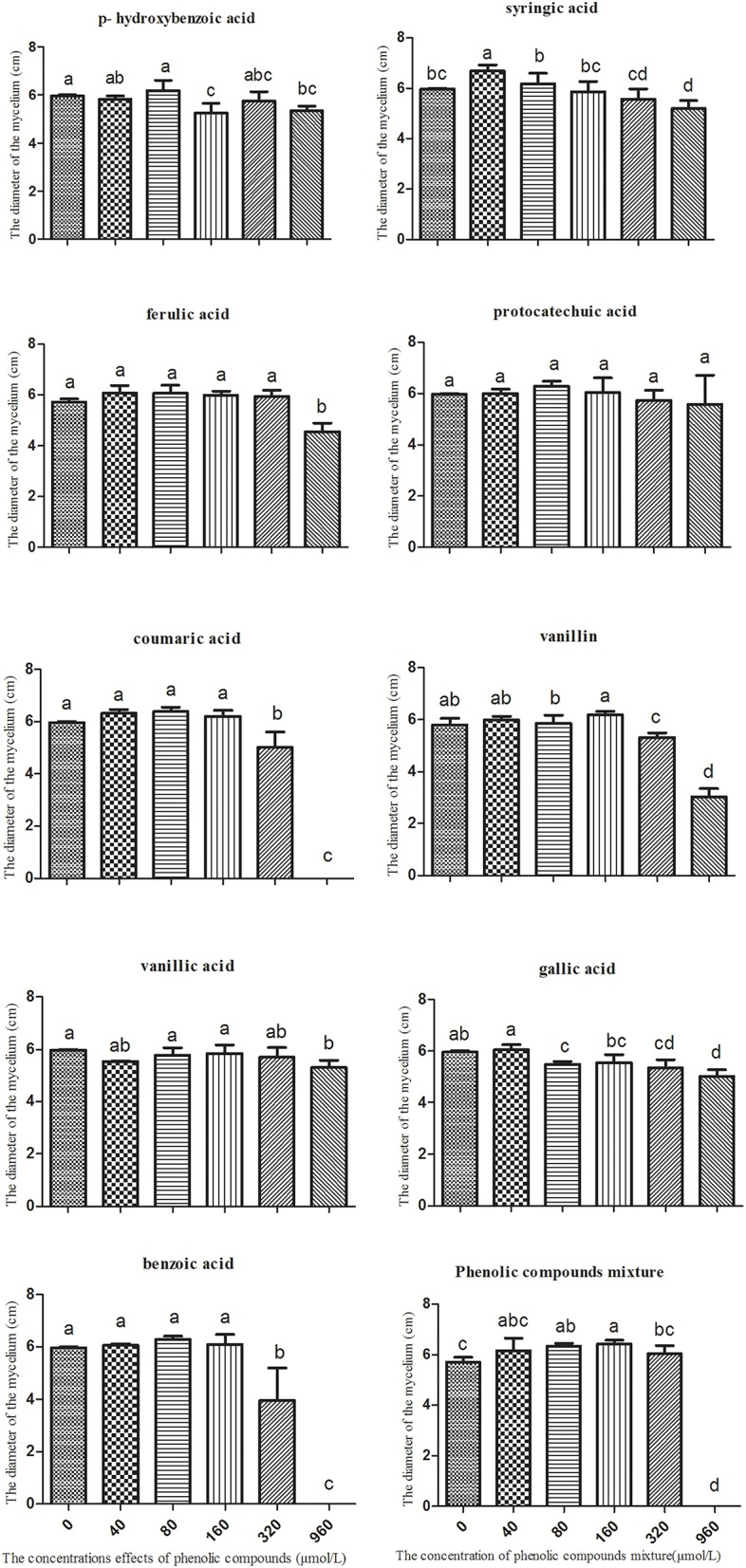
**The effects of single phenolic compounds and their mixture on the mycelial growth of *Talaromyces helices* M**. Columns with different letters are statistically different (LSD-test, *p* < 0.05).

Mixed phenolic acids significantly promoted the mycelial growth of *T. helices*. The greatest increase occurred when exposed to the dosage at 160 μmol/L of the mixed phenolic acids (Figure 4). All treatment concentrations of phenolic acids in the mixture had a positive effect on mycelial growth of *T. helicus*. The various single phenolic acids had different influences on the mycelial growth. Syringic acid had the greatest positive effect on the *T. helicus* at 40 μmol/L, whereas vanillin and gallic acid showed variable effects, with a growth promotion effect at a low concentration and a suppressive effect at a high concentration. Hydroxybenzoic acid, ferulic acid, protocatechuic acid, coumaric acid, vanillic acid, and benzoic acid had no significant effect on *T. helicus* at a low concentration, but they had an inhibitory effect at high concentrations (Figure 4). We suggest that not all of the individual phenolic acids can boost the growth of *T. helices*. Some phenolic acids showed an inhibitory effect. However, the phenolic acids mixed in the same ratio as the result of HPLC analysis for monocultured *R. pseudostellariae* rhizosphere soil significantly promoted the growth of the pathogenic *T. helicus* strains.

**Figure 5:**
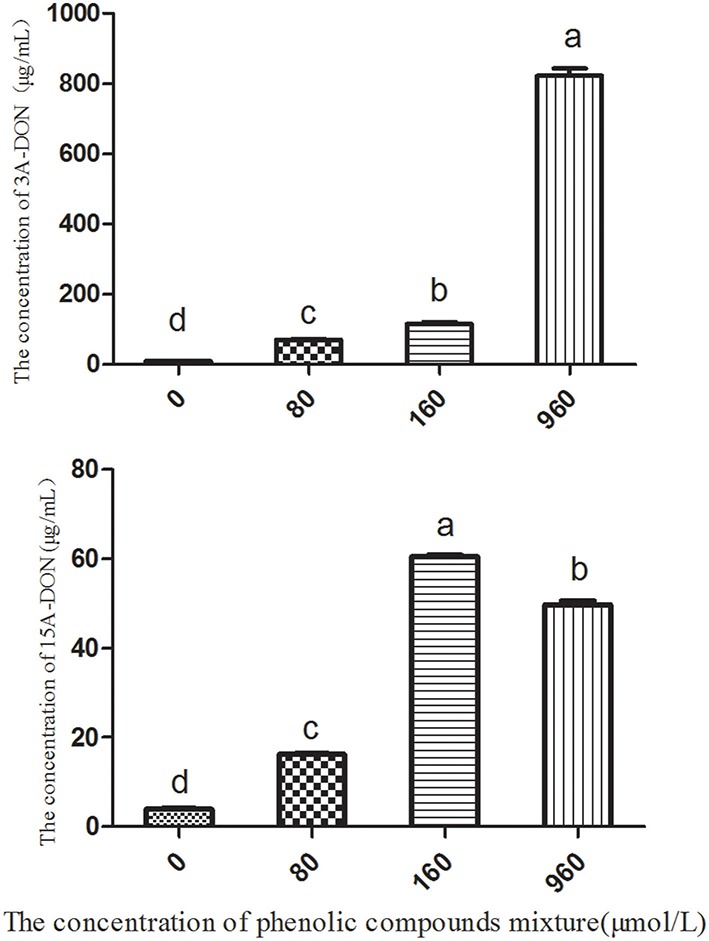
**The effects of the phenolic compound mixture on the toxin production of *T. helicus***. Columns with different letters are statistically different (LSD-test, *p* < 0.05).

The soil extracts, which were diluted 10 times and used as the culture medium for *T. helicus*, contained two types of toxins, 3A-DON and 15A-DON. The concentration of 3A-DON was significantly higher than that of 15A-DON (Table 1 and Figure S9). However, the production of the 15A-DON toxin can be promoted by *T. helicus* when exposed to increasing dosages of the mixed phenolic acids, and the toxin content increased sharply, then reached its highest value at 160 μmol/L. The 15A-DON toxin levels were also greater than those in the control group (0 μmol/L). The production of 3A-DON also increased with an increase in the treatment concentration of the mixed phenolic acids. This suggests that the mixed phenolic acids were favorable for the mycelial growth and toxin production of the pathogenic bacteria, such as *T. helicus* (Figure 5).

**Figure 6:**
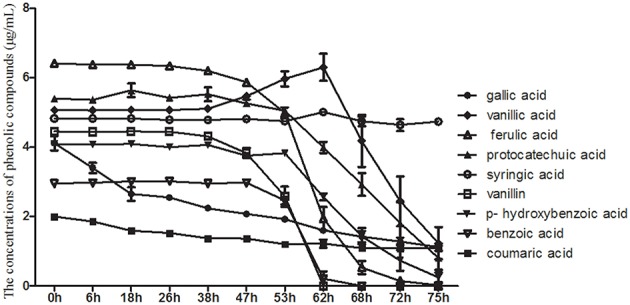
**The consumption of phenolic compounds by *Talaromyces* M**.

HPLC was used to detect the use of nine phenolic acids by the pathogenic fungus, *T. helices*, which was detected in the rhizosphere soil of the monocultured *R. pseudostellariae*. The results showed that *T. helicus* can use eight types of phenolic acids, i.e., gallic acid, coumaric acid, protocatechuic acid, p-hydroxy benzoic acid, vanillic acid, vanillin, ferulic acid, and benzoic acid (Figure 6). However, syringic acid, which had a stimulatory effect on the pathogenic fungus at a low treatment concentration but an inhibitory effect at a high concentration, could not be used although a stimulatory effect was observed on the mycelial growth of *T. helicus* at treatment concentrations of more than 40 μmol/L.

### The influence of phenolic acids on the physiological characteristics of *K. sacchari*

**Figure 7:**
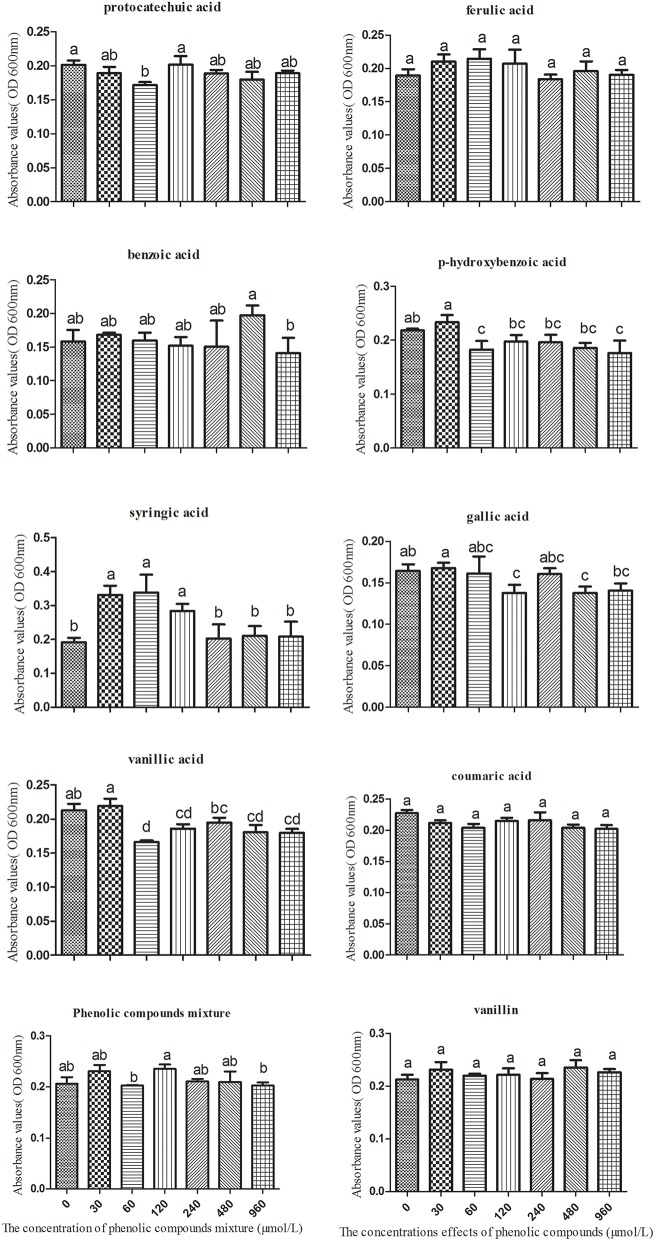
**The effects of single phenolic compounds and the phenolic compound mixture on the growth of *Kosakonia sacchari***. Columns with different letters are statistically different (LSD-test, *p* < 0.05).

Based on the molar proportions of the phenolic acids in the soil, we made a series of mixtures with eight types of phenolic acids at concentrations of 0, 30, 60, 120, 240, 480, and 960 μmol/L. These were used to analyze the impact on the growth of *K. sacchari*. Different concentrations of the mixed phenolic acids had different effects on *K. sacchari* compared with *T. helices*. Low concentrations of the phenolic acid mixture had a stimulatory effect on specific pathogens, whereas the reverse was true at high treatment concentrations. Mixed phenolic acids promote the growth of *K. sacchari*, but only within a specific range of concentrations. Each phenolic acid had distinct effects on the growth of *K. sacchari*. Syringic acid had the greatest effect on the pathogenic bacterium at a treatment concentration of 60 μmol/L. Gallic acid, p-hydroxybenzoic acid, vanillic acid, and benzoic acid promote growth at low concentrations but suppress it at high concentrations. Coumaric acid, protocatechuic acid, vanillin, and ferulic acid had no significant effect at low concentrations (Figure 7). Thus, the main allelochemicals of *R. pseudostellariae* root exudates could have distinctly positive or negative effects on the growth of pathogenic fungi and bacteria, implying that root exudates can exert either positive or negative selection on specific microbes.

**Figure 8:**
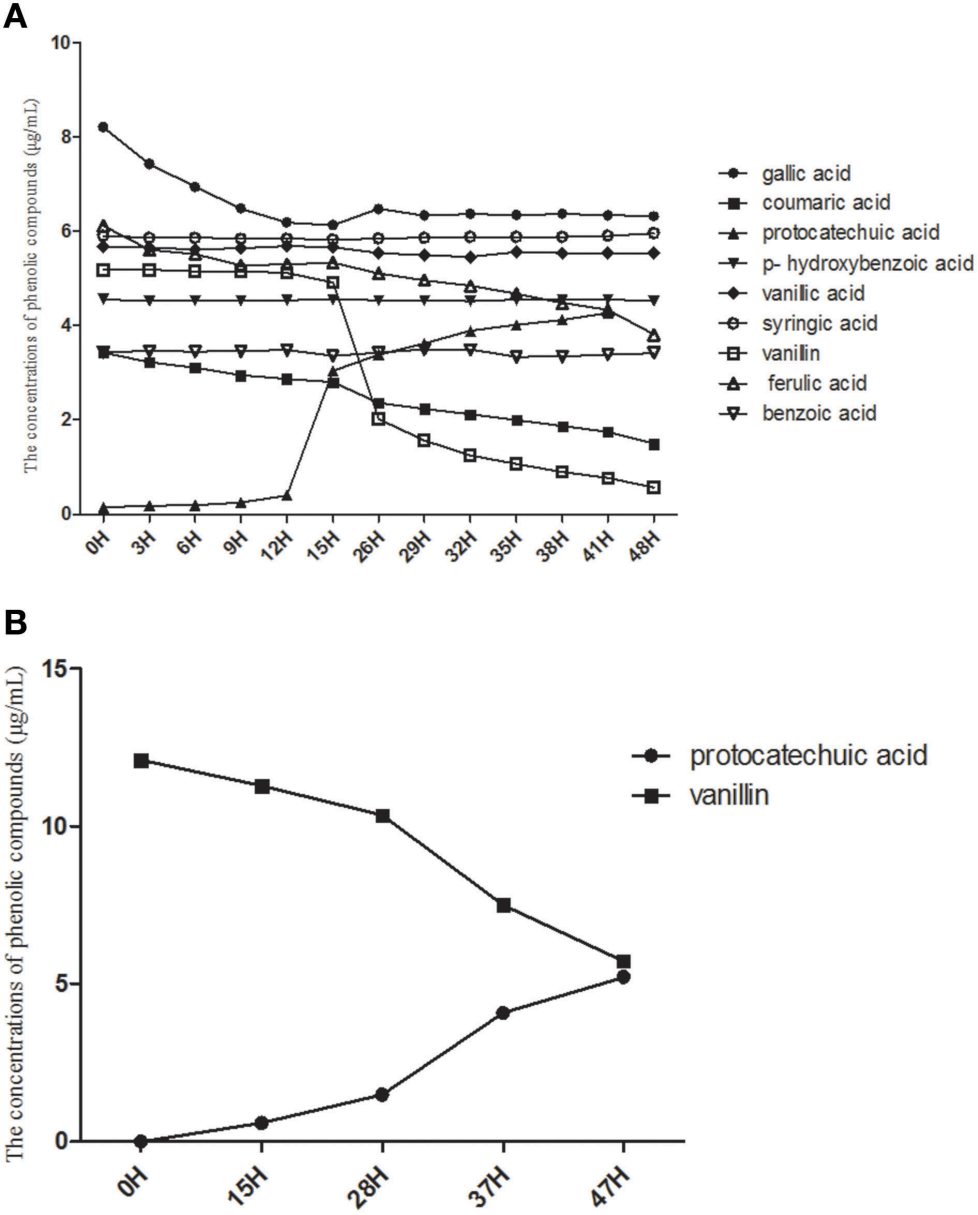
**The consumption of phenolic compounds (A) and single vanillin (B) by *Kosakonia* W**.

Based on the consumption of eight phenolic acids detected in the soil, we found that *K. sacchari* could only use four types, i.e., gallic acid, coumaric acid, vanillin, and ferulic acid (Figure 8). The utilization efficiency of vanillin was the highest of the four compounds. *K. sacchari* is able to produce protocatechuic acid from consumption of vanillin (Figure 8).

### The influence of toxin production and protocatechuic acid on the growth of *K. sacchari* and *B. pumilus*

**Figure 9:**
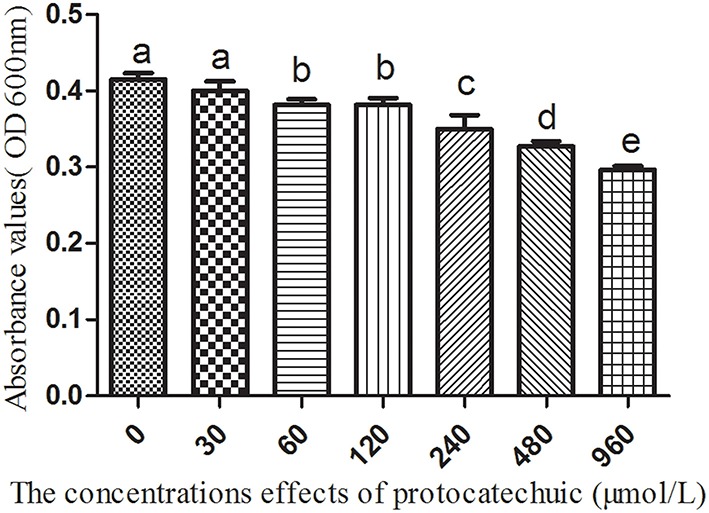
**The effects of protocatechuic acid on the growth of *Bacillus pumilus* Z**. Columns with different letters are statistically different (LSD-test, *p* < 0.05).

The 3A-DON toxin, at a low concentration promoted the growth of *K. sacchari* and inhibited the growth of *B. pumilus*. In contrast, the 15A-DON toxin had no significant effect on their growth at a low treatment concentration (Figures S10, S11). Protocatechuic acid, at 60 μmol/L, had a negative effect on the growth of the beneficial *B. pumilus* Z. (Figure 9).

## Discussion

Previous research related to plant allelopathy or the negative effects of continuous monoculture mainly focused on isolation, identification, and bioassay of allelochemicals, and the evaluation of the direct influence of allelochemicals on the growth of receptor plants (Li et al., 2012). However, the composition and effective concentration of allelochemicals remains controversial. Some believe that the effective concentration of phenolic allelochemicals that can inhibit weeds or stunt crop growth is much higher than that found under field conditions. It is now known that allelochemicals released into the soil can be catabolized, transformed, and processed by microorganisms (Eisenhauer et al., 2012). Allelopathy can operate indirectly between the donor and the recipient plant or between crop residue and new plantings (Kato, 2009; Lin, 2013). Positive or negative effects of plant-plant interactions, such as plant allelopathy, allelopathic autotoxicity, intercropping benefits, or exotic plant invasion, can result from interactions between plants and specific microorganisms, which are often mediated by root exudates (Wang et al., 2013). Plants can transport the carbon fixed by photosynthesis to below ground, where it is released into the soil and provides a source of carbon and energy for microbial growth. Simultaneously, the changes in microbial community structure will affect plant growth both below and above ground (Wardle et al., 2004). Lin et al. (2011) added major rice allelochemicals (p-hydroxy benzoic acid, ferulic acid, salicylic acid, vanillic acid, cinnamic acid) to the soil and found that these phenolic acids decreased by approximately 50–90% from 3 to 7 d. This may be related to microbial decomposition and utilization.

**Figure 10:**
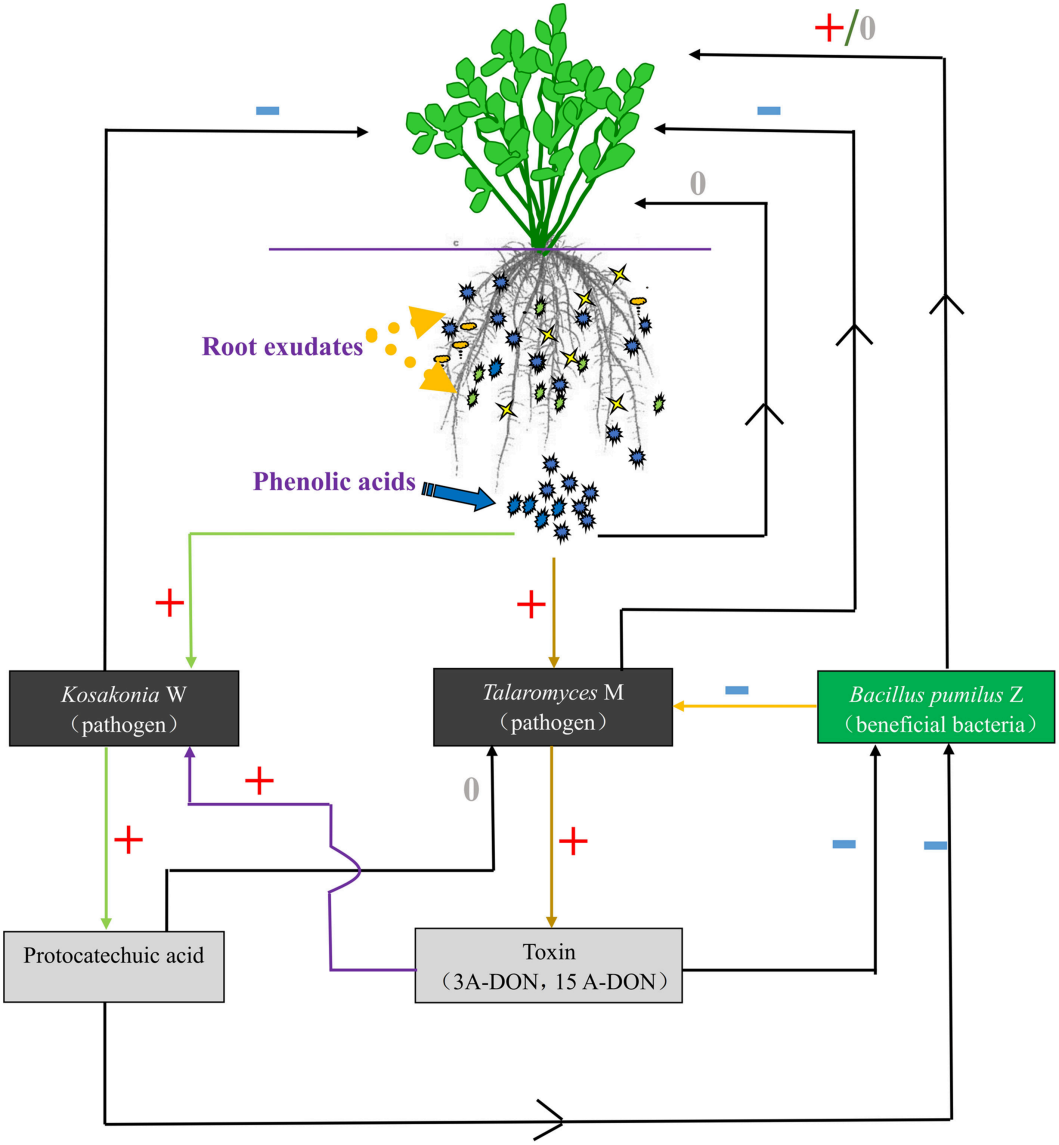
**The communication between plants and microorganisms**. +, Positive effect; −, negative effect; 0, no effect.

In the present study, we found that many of the phenolic acids produced by tissue culture plantlet roots of *R. pseudostellariae* accumulated in the culture medium and showed no autotoxicity over the period of plantlet growth (Figure S8). However, these phenolic acids did not increase over the years of monoculture and they did not accumulate in the rhizosphere soil. What is more, the levels of some phenolic acids detected in consecutively monocultured soil were lower than the levels in normal cropping soil, and most of the concerned phenolic acids increased initially and then declined after a several years of continuous cropping. In addition, the content of all the phenolic acids concerned was variable among the different growth stages due to secretions of root exudates, decomposition, synthesis, and transformation of allelochemicals by soil microbes in different monoculture systems. This findings suggest that the consecutive monoculture problems are not due to the direct effects of high phenolic acid concentrations on the receptor plants, but more likely related to indirect effects on plant growth caused by changing microbial flora as shown in Figure 10. Zhou and Wu (2012b) found that after 7 years of continuous cucumber cropping, the plant biomass had declined to the lowest level in the study. However, after 7th year of monocropping, the plant biomass gradually increased. The content of total soil phenolics and some phenolic acids (such as ferulic acid, p-hydroxybenzoic, p-coumaric acid, etc.) showed a similar trend, i.e., the content was lowest in the 7th year, after which it gradually increased. This may be the reason that the most serious disease problems result from the rhizosphere interaction involved in shifts of microbial community differentially mediated by root exudates in monocropping system.

*T. helices* and *K. sacchari* used in this study were validated as important soil-borne pathogens. We found different phenolic acids differentially mediate the proliferation of the two targets. *T. helices* and *K. sacchari* cannot directly utilize syringic acid as carbon or nitrogen sources. But they may play an important role in promoting the growth of pathogenic microorganisms in the rhizosphere soil of *R. pseudostellariae*. Many studies (Zhang et al., 2008; Wei et al., 2010) have demonstrated that syringic acid functioning as a signal molecule, promotes cell proliferation and regulates the cell cycle. Some researchers also reported that the *K. sacchari* was isolated from infected stem, root or rhizosphere soil of sugar cane (Zhu et al., 2013; Gu et al., 2014). We have used the quantitative real-time PCR to detect it in the rhizosphere soil of *R. pseudostellariae*. The result showed a significant increase in the amount of pathogenic *K. sacchari* in the rhizosphere of *R. pseudostellariae* as the number of monoculture years increased, especially around the site of infected *R. pseudostellariae* (SS). *K. sacchari* was highly pathogenic to the tissue culture plantlets of *R. pseudostellariae* (Figure S1). And the pathogen could also affect other beneficial bacteria population such as *B. pumilus* used in this study via its toxin production and its metabolic intermediate. Our results demonstrated that, *K. sacchari* was able to utilize root exudate, vanillin to produce an intermediate product, protocatechuic acid (PA), which was considered as a better antibacterial substance in previous studies. We also found that PA did not suppress the growth of the pathogenic *K. sacchari*, but it did have negative effects on the growth of the beneficial, *B. pumilus* at a low concentration. *B. pumilus* is a PGPR, which has been successfully used for the biological control of damping-off diseases (Huang et al., 2012). Tadych et al. (2015) also proposed quinic acids could significantly influence virulent facctors of the fruit rot fungi. In our studies, the 3A-DON toxin production of the pathogenic, *T. helices*, can be promoted by some phenolic acids, especially by the phenolic acids mixed in the same ratio as detected in monocultured rhizosphere soil, then a cascade reaction was triggered off to promote the growth of the other pathogenic fungus, *K. sacchari* and inhibited the growth of its counterpart, the beneficial bacterium, *B. pumilus*, (Figure S4). An increase in populations of soil-borne pathogenic fungi (e.g., *Fusarium oxysporum*) is likely responsible for soil sickness (Qi et al., 2009), but the present study also confirmed that the two soil-borne pathogenic microorganisms were also responsible for the monoculture problems. Therefore, it is very complicated by the fact that monoculture problems are involved in an intricate rhizosphere interaction. The imbalanced population structure, with the higher population sizes of *T. helices* and *K. sacchari* but lower *B. pumilus* population influenced by the mixed phenolic acid exudates, may partially account for the soil sickness of *R. pseudostellariae*.

Our results unveil the underlying mechanism of replanting diseases of *R. pseudostellariae* popularly in modern agriculture system, especially in nowadays China. The case study deeply illustrates about how root exudates play important roles in the formation of monoculture problems involved in rhizosphere interactions under monocropping regimes. As we know, root exudates are carbon sources for soil microbial growth and also signal substances that function as communication tools between plants and microorganisms in the rhizosphere (Mandal et al., 2010). Peters et al. (1986) found that expression of the nodulation gene (nodD) in nodule bacteria is directly related to legume root secretions of flavonoids and isoflavonoids. Venkatachalam et al. (2012) showed that tomatoes whose leaves were infected by pathogens were able to attract more beneficial bacteria such as *B. subtilis* to the rhizosphere by regulating root secretion. This was accomplished by increasing the synthesis and release of malic acid, leading to induced systemic resistance allowing plants to better defend against further pathogen infestation. Rousk et al. (2010) also found the pH gradients could change the diversity of the bacterial and fungal communities in an arable soil. Root exudates play an important role in the interaction between plants and microbes. They can have different effects on different microbes, which include both stimulatory and inhibitory influences. For example, corn root secretion of DIMBOA (DIMBOA, 2,4-dihydroxy-7-methoxy-2H-1, 4-benzoxazin-3 (4H)-one) and *Ocimum basilicum* L. secretion of rosmarinic acid resulted in strong antibacterial activity (Bais et al., 2006; Berendsen et al., 2012). Zhou et al. (2012) found that the allelopathic autotoxicity of coumaric acid from cucumber can significantly promote the growth of the soil pathogen *F. oxysporum*. Others have found that root exudates of maize, peanuts, watermelons, and American ginseng can significantly promote the growth of pathogens, leading to increased soil-borne disease (Ju et al., 2002; Li et al., 2009; Hao et al., 2010). Benizri et al. (2005) showed that the number of beneficial bacteria was reduced and the number of pathogenic bacteria was increased in continuously cropped peach soil. Our results show the same phenomenon, which is attributed to the changes in soil microbial composition and structure differentially mediated by phenolic acids of root exudates.

This findings provide a clue to open a new avenue for modulating the root microbiome to enhance medicinal plant production and sustainability. Such approaches might include the use of microbial fertilizer and organic amendment application to keep the balance of microbial community in rhizosphere soil in modern agriculture system. And the amendment of the root exudates in *R. pseudostellariae* can also be used to relieve the problems. Additional work is needed to further understand the intrinsic mechanism of these specific microbial functions in the future.

## Funding

This work was supported by the National Science Foundation of China (Grant no. U1205021, 81573530, 81303170, 31401950, 31301858), the National Key Basic Research Program of China (Grant no. 2012CB126309), and the Health and Family planning Program of Fujian province (Grant no. WKJ-FJ-34).

### Conflict of interest statement

The authors declare that the research was conducted in the absence of any commercial or financial relationships that could be construed as a potential conflict of interest.
