# Supplementary material for: Mixed Phenolic Acids Mediated Proliferation of Pathogens Talaromyces helicus and Kosakonia sacchari in Continuously Monocultured Radix pseudostellariae Rhizosphere Soil
Source: Front Microbiol. 2016 Mar 17;7:335. doi: 10.3389/fmicb.2016.00335 (PMC4795122; doi:10.3389/fmicb.2016.00335)
Supplement: Supplementary file 1 [file Table1.PDF]

Supplementary Material

Mixed phenolic acids mediated proliferation of pathogens *Talaromyces helicus* and *Kosakonia sacchari* in continuously monocultured *Radix pseudostellariae* rhizosphere soil

Hongmiao Wu, Linkun Wu, Juanying Wang, Quan Zhu, Sheng Lin, Jiahui Xu, Cailiang Zheng, Jun Chen, Xianjin Qin, Changxun Fang, Zhixing Zhang, Saadia Azeem, Wenxiong Lin*

Corresponding author: Dr. Wenxiong Lin
E-mail: wenxiong181@163.com

Supplemental Table S1 DNA sequences of the primer pairs used in this study

Primer	Sequence	References
1405f	5' TGYACACACCGCCCGT 3'	Zeng *et al.*, 2011
456r	5' CCTTTCCCTCACGGTACTG3'	
ITS4	5'TCCTCCGCTTATTGATATGC 3'	Consuelo *et al.*, 2001
ITS86	5'GTGAATCATCGAATCTTTGAAC3'	
TH1F	5' TGATCCGAGGTCAACCGTAA 3'	This study
TH1R	5' CCTGTCCGAGCGTCATTTCT 3'	
saesu-F	5'TTCATCGCCTCTGACTGC3'	This study
saesu-R	5'GCGTTGCCAACCGTATCT 3'	
BP-F	5' TCGGTGTTCGTCCCGTCCTT 3'	This study
BP-R	5'GCGTGCGTTTTCCTCTTGTTTAGT 3'	

Supplemental Table S2 EC50 values of phenolic acids on the *Kosakonia sacchari*, *Talaromyces helices* and *Bacillus pumilus*

	EC50 of *Kosakonia sacchari* (μmol/L)	EC50 of *Talaromyces helices* (μmol/L)	EC50 of *Bacillus pumilus* (μmol/L)
Gallic acid	7200	1800	/
Coumaric acid	2400	960	/
Protocatechuic acid	5200	2400	2400
P-hydroxybenzoic acid	3600	1800	/
Vanillic acid	7200	1800	/
Syringic acid	7200	1800	/
Vanillin	10000	1200	/
Ferulic acid	5200	1200	/
Benzoic acid	3600	960	/
Phenolic compound mixture	12000	960	/
