# Supplementary material for: Mixed Phenolic Acids Mediated Proliferation of Pathogens Talaromyces helicus and Kosakonia sacchari in Continuously Monocultured Radix pseudostellariae Rhizosphere Soil
Source: Front Microbiol. 2016 Mar 17;7:335. doi: 10.3389/fmicb.2016.00335 (PMC4795122; doi:10.3389/fmicb.2016.00335)
Supplement: Supplementary file 2 [file DataSheet1.pdf]

Supplementary Material

Mixed phenolic acids mediated proliferation of pathogens *Talaromyces helicus* and *Kosakonia sacchari* in continuously monocultured *Radix pseudostellariae* rhizosphere soil

Hongmiao Wu, Linkun Wu, Juanying Wang, Quan Zhu, Sheng Lin, Jiahui Xu, Cailiang Zheng, Jun Chen, Xianjin Qin, Changxun Fang, Zhixing Zhang, Saadia Azeem, Wenxiong Lin*

Corresponding author: Dr. Wenxiong Lin
E-mail: wenxiong181@163.com

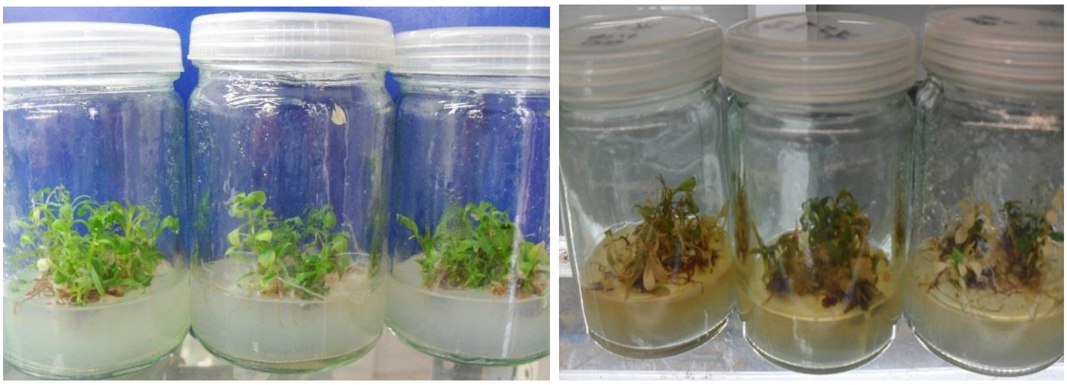

Supplemental Figure S1 The validation of pathogenicity of tissue-cultured plantlets of *Radix pseudostellariae* infected by *Kosakonia sacchari* W.

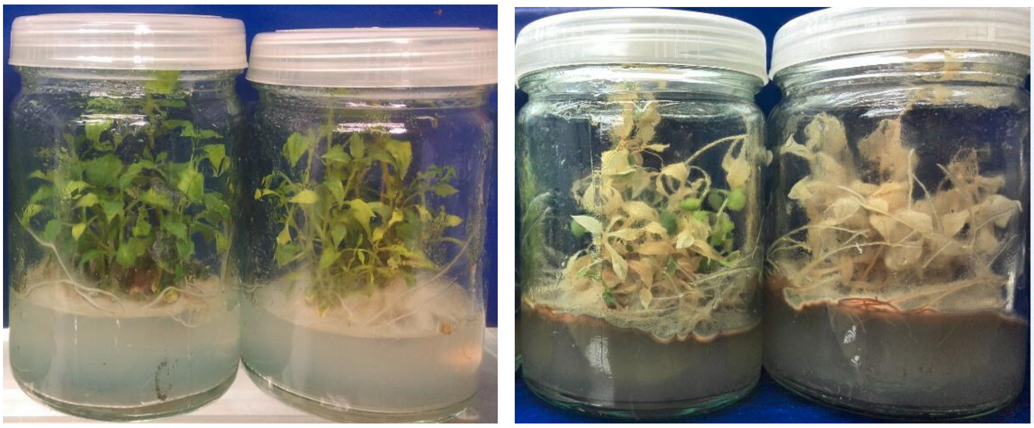

Supplemental Figure S2 The validation of pathogenicity of the tissue-cultured plantlets of *Radix pseudostellariae* infected by *Talaromyces helices* M.

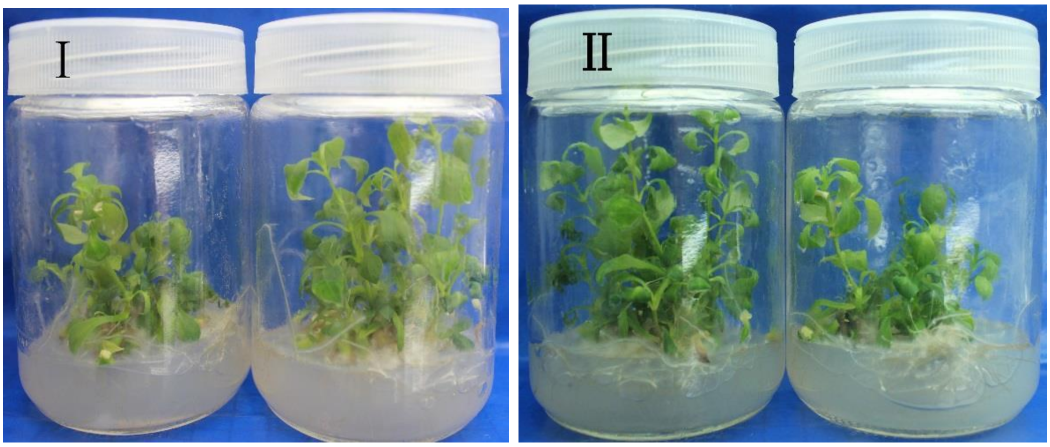

Supplemental Figure S3 The validation of pathogenicity of the tissue-cultured plantlets of *Radix pseudostellariae* infected by *Bacillus pumilus Z.* I: before adding *Bacillus pumilus* Z., II: 30 days later.

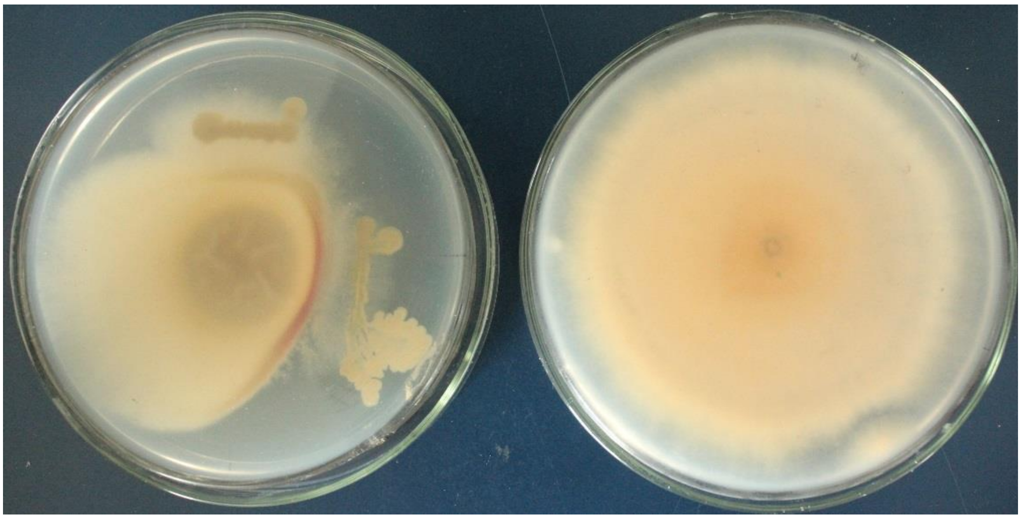

Supplemental Figure S4 Agar disk diffusion method used to screen the *Bacillus pumilus* Z. antagonists against *Talaromyces* M.

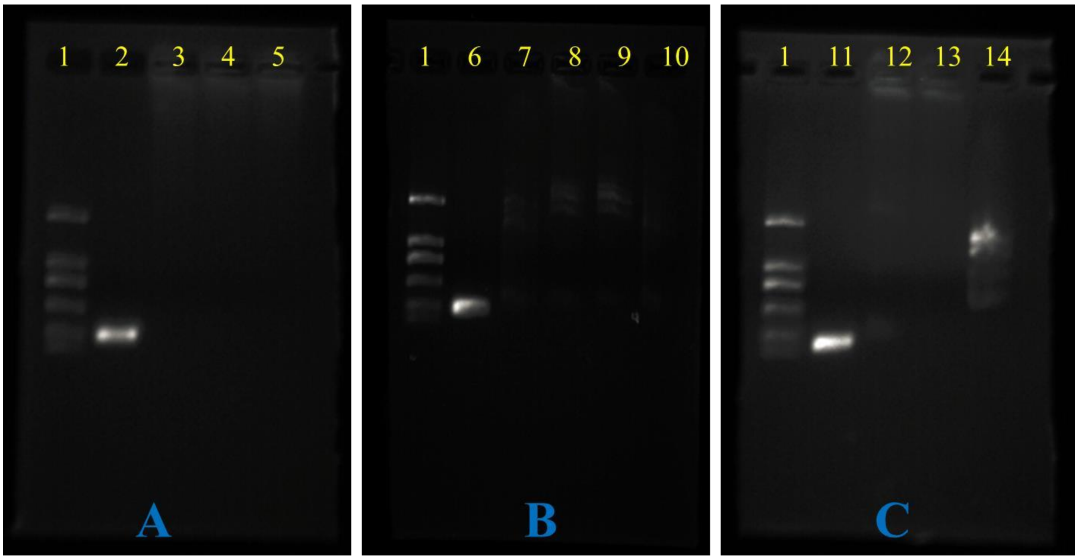

Supplemental Figure S5 The PCR validation test of specific primer. A: The test for *Bacillus pumilus* Z; B: The test for *Kosakonia sacchari* W; C: The test for *Talaromyces helicus* M. 1: DL2000 DNA Marker; 2,7,14: *Bacillus pumilus* Z; 3,6: *Kosakonia sacchari* W; 4,10: *Bacillus megaterium*; 5,12: *Fusarium solani*; 8,13: *Fusarium oxysporum*; 9,11: *Talaromyces helicus* M.

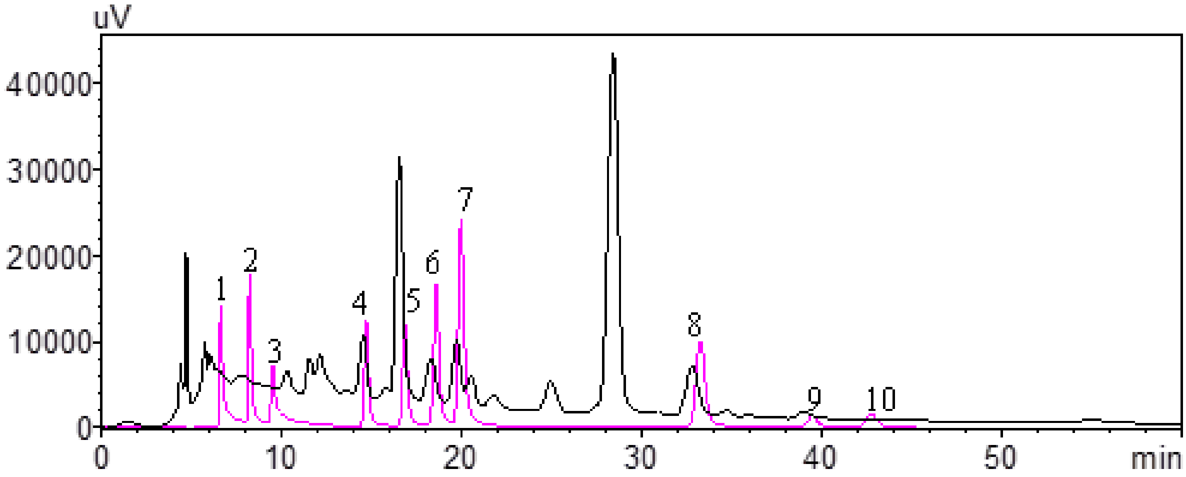

Supplemental Figure S6 The chromatogram of phenolic compounds detected by HPLC. Red chromatogram means the standard compounds; Black chromatogram means the sample. 1 represents gallic acid; 2 represents coumaric acid; 3 represents protocatechuic acid; 4 represents p-hydroxybenzoic acid; 5 represents vanillic acid; 6 represents syringic acid; 7 represents vanillin; 8 represents ferulic acid; 9 represents benzoic acid; 10 represents salicylic acid.

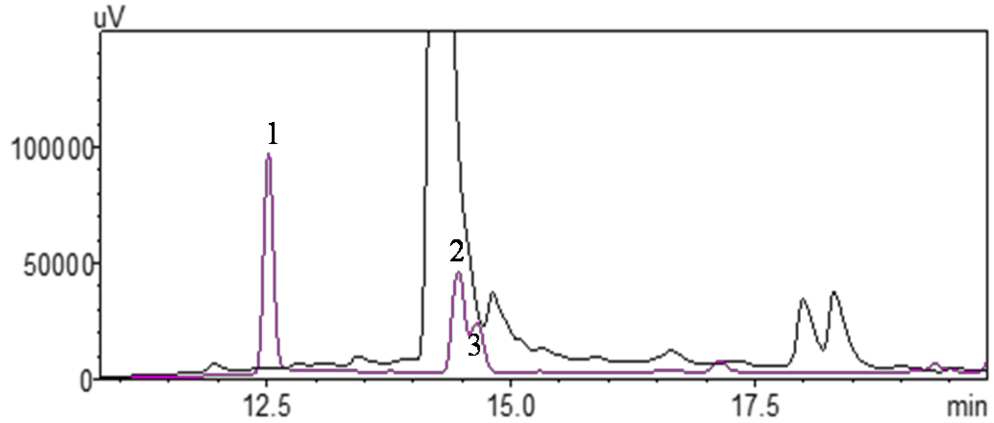

Supplemental Figure S9 The chromatogram of toxins detected by HPLC. Red chromatogram means the standard compounds; Black chromatogram means the sample. 1 represents DON; 2 represents 3A-DON; 3 represents 15A-DON.
