# Supplementary material for: Mixed Phenolic Acids Mediated Proliferation of Pathogens Talaromyces helicus and Kosakonia sacchari in Continuously Monocultured Radix pseudostellariae Rhizosphere Soil
Source: Front Microbiol. 2016 Mar 17;7:335. doi: 10.3389/fmicb.2016.00335 (PMC4795122; doi:10.3389/fmicb.2016.00335)
Supplement: Supplementary file 3 [file DataSheet2.pdf]

Supplementary Material

Mixed phenolic acids mediated proliferation of pathogens *Talaromyces helicus* and *Kosakonia sacchari* in continuously monocultured *Radix pseudostellariae* rhizosphere soil

Hongmiao Wu, Linkun Wu, Juanying Wang, Quan Zhu, Sheng Lin, Jiahui Xu, Cailiang Zheng, Jun Chen, Xianjin Qin, Changxun Fang, Zhixing Zhang, Saadia Azeem, Wenxiong Lin*

Corresponding author: Dr. Wenxiong Lin
E-mail: wenxiong181@163.com

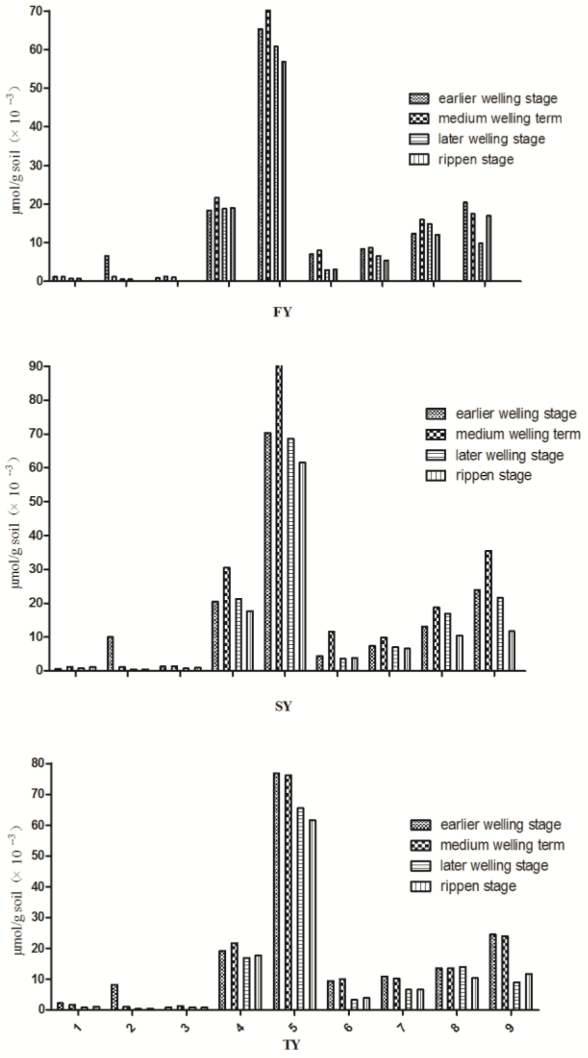

Supplemental Figure S7 Changes in the levels of phenolic compounds in the rhizosphere soil of *Radix pseudostellariae* in a continuous cropping system sampled at different growth stages. 1 represents gallic acid; 2 represents coumaric acid; 3 represents protocatechuic acid; 4 represents p-hydroxybenzoic acid; 5 represents vanillic acid; 6 represents syringic acid; 7 represents vanillin; 8 represents ferulic acid; 9 represents benzoic acid; FY, SY, TY represent the first, second, and third *Radix pseudostellariae* cropping, respectively.

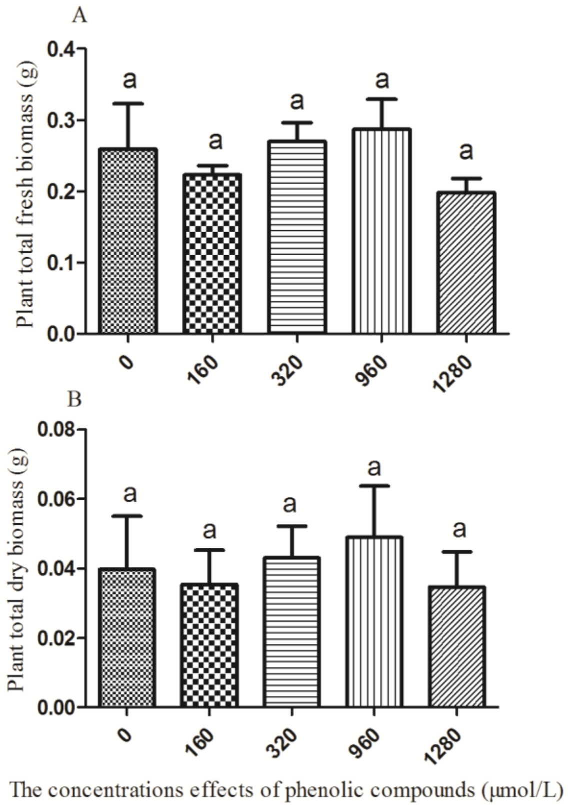

Supplemental Figure S8 The effects of phenolic compounds on *Radix pseudostellariae* total fresh biomass **(**A**)** and dry biomass **(**B**)** in tissue culture medium. Columns with different letters are statistically different (LSD test, $p < 0.05$).

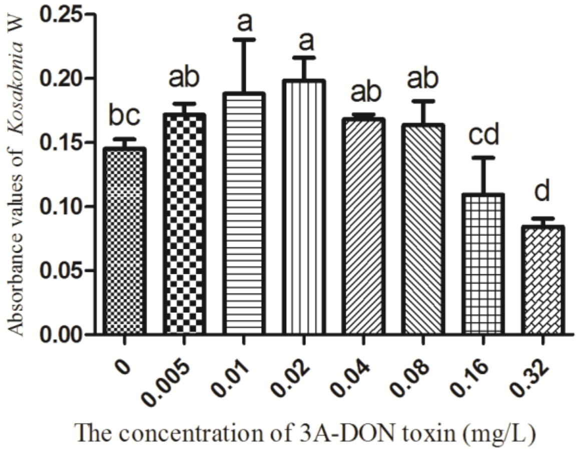

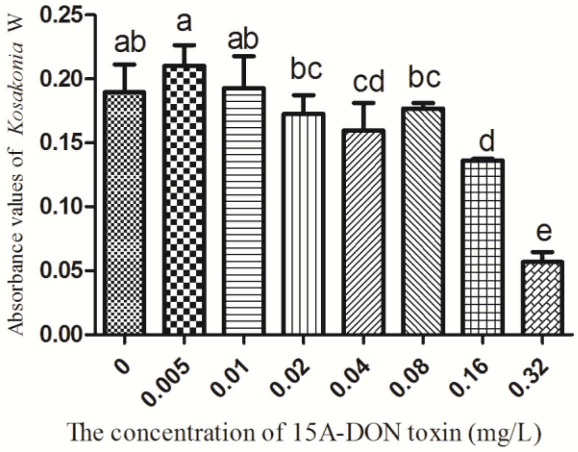

Supplemental Figure S10 The effects of toxins on the growth of *Kosakonia sacchari* W. Columns with different letters are statistically different (LSD test, $p < 0.05$).

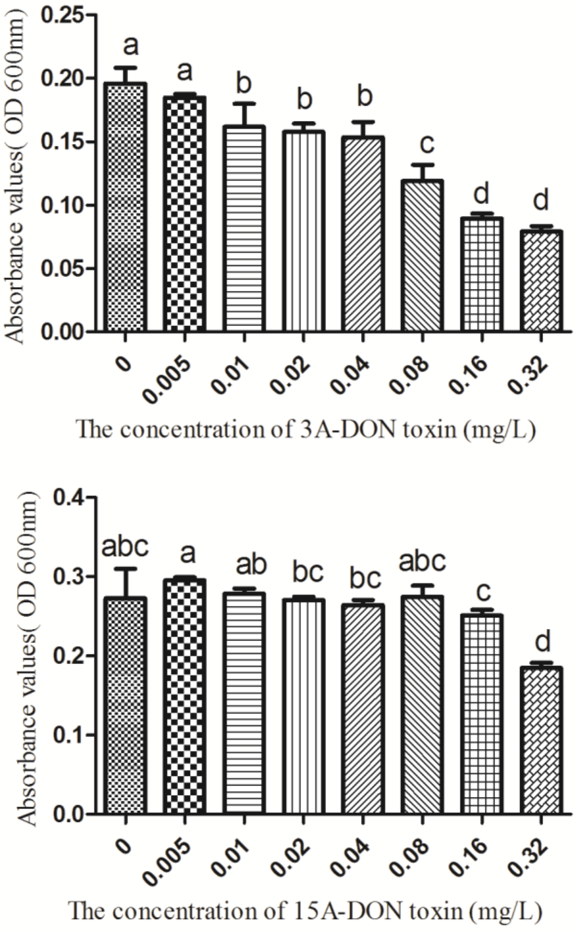

Supplemental Figure S11 The effects of toxins on the growth of *Bacillus pumilus* Z. Columns with different letters are statistically different (LSD test, $p < 0.05$).
